# Superficial Temporal Artery-to-Middle Cerebral Artery Bypass in Ischemic Stroke With Blood Pressure-Dependent Symptoms

**DOI:** 10.7759/cureus.56236

**Published:** 2024-03-15

**Authors:** Brendan Huang, Calvin Huang, Khaled Alok, Alex Y Chen

**Affiliations:** 1 Neurology, Northwell Health, Manhasset, USA; 2 Neurosurgery, Northwell Health, Manhasset, USA

**Keywords:** ec-ic bypass, intracranial atherosclerosis, nihss score, sta-mca bypass, ischemic stroke, ct brain perfusion

## Abstract

The efficacy of extracranial-intracranial (EC-IC) bypass in preventing ischemic stroke progression and recurrence is controversial. As per the current hypothesis, EC-IC bypass is most beneficial for patients with persistent hemodynamic insufficiency. Hence, various approaches have been used to evaluate hemodynamic insufficiency, including repeated single photon emission CT (SPECT) imaging or continuous monitoring of cerebral flow with transcranial Doppler ultrasound (TCD). However, both modalities are time- and resource-intensive. In this report, we discuss how EC-IC bypass turned out to be beneficial for a patient presenting with blood pressure-dependent severe aphasia and right hemiparesis due to middle cerebral artery (MCA) occlusion that failed thrombectomy. CT perfusion (CTP) scan at admission demonstrated a persistent volume of delayed perfusion without core infarct. Following the superficial temporal artery‐to‐middle cerebral artery (STA-MCA) bypass, the patient's National Institute of Health Stroke Scale (NIHSS) score improved from 12 to 1. Ischemic penumbra, as seen on CTP imaging, also improved after the STA-MCA bypass. Our case suggests that persistent volume of delayed perfusion and blood pressure-dependent neurological deficits can be used in tandem as selection criteria for EC-IC bypass.

## Introduction

Intracranial atherosclerosis with moderate to high-level stenosis (70-99%) is a leading risk factor for stroke [1]. Asians have a higher risk of ischemic stroke secondary to intracranial atherosclerosis when compared to Caucasians, [2]. Several ongoing trials such as CHANCE-2 and CAPTIVA have attempted to elucidate the optimal medical management for stroke patients with intracranial atherosclerosis mechanism [3,4]. Despite undergoing medical management, patients with intracranial atherosclerosis remain at a higher risk for recurrent ischemic strokes [5].

The direct extracranial-intracranial (EC-IC) bypass is a procedure whereby a large extracranial artery is anastomosed to an intracranial artery to restore blood flow into the brain. A common approach involves using the superficial temporal artery‐to‐middle cerebral artery (STA-MCA) bypass to directly augment cerebral flow for patients with severe stenosis or acute occlusion of the internal carotid artery (ICA) or MCA in the setting of acute ischemic stroke. The goals of the procedure are to prevent stroke progression, which often manifests as worsening National Institute of Health Stroke Scale (NIHSS) or modified Rankin scale (mRS) scores, and to prevent recurrent ischemic stroke. EC-IC bypass initially failed to show benefit in the 1985 EC/IC Bypass Study Group [6]. In this study, patients with transient ischemic stroke (TIA) or mild acute ischemic stroke (AIS) with occlusion of ICA and/or MCA were included. Later, COSS and RECON trials also failed to show the benefits of bypass [7,8]. However, neither study selected patients with ongoing hemodynamic symptoms or persistent brain tissue at risk of infarction [9].

More recently, EC-IC bypass has been found to improve cerebral hemodynamic parameters and reduce subsequent cerebral ischemic events by over 50% [10]. Those patients were selected using continuous transcranial Doppler ultrasound (TCD) monitoring with and without voluntary breath-holding and repeated single photon emission CT (SPECT) imaging paired with acetazolamide-challenge. While both modalities are specifically used for selecting patients who would likely benefit from EC-IC bypass, they are rarely used in practice due to their time- and resource-intensive nature [11]. We report a case of a patient who presented with episodic stroke symptoms that were blood pressure-dependent and in whom a CT perfusion (CTP) scan demonstrated persistent ischemic brain tissue. After the STA-MCA bypass, the patient's NIHSS score improved at discharge and the patient demonstrated no recurrent cerebral event at the six-month follow-up. Our case endorses the concept that relying on both blood pressure-dependent stroke symptoms and persistent penumbra on CTP can be a pragmatic approach for selecting patients for EC-IC bypass.

## Case presentation

**Figure 1:**
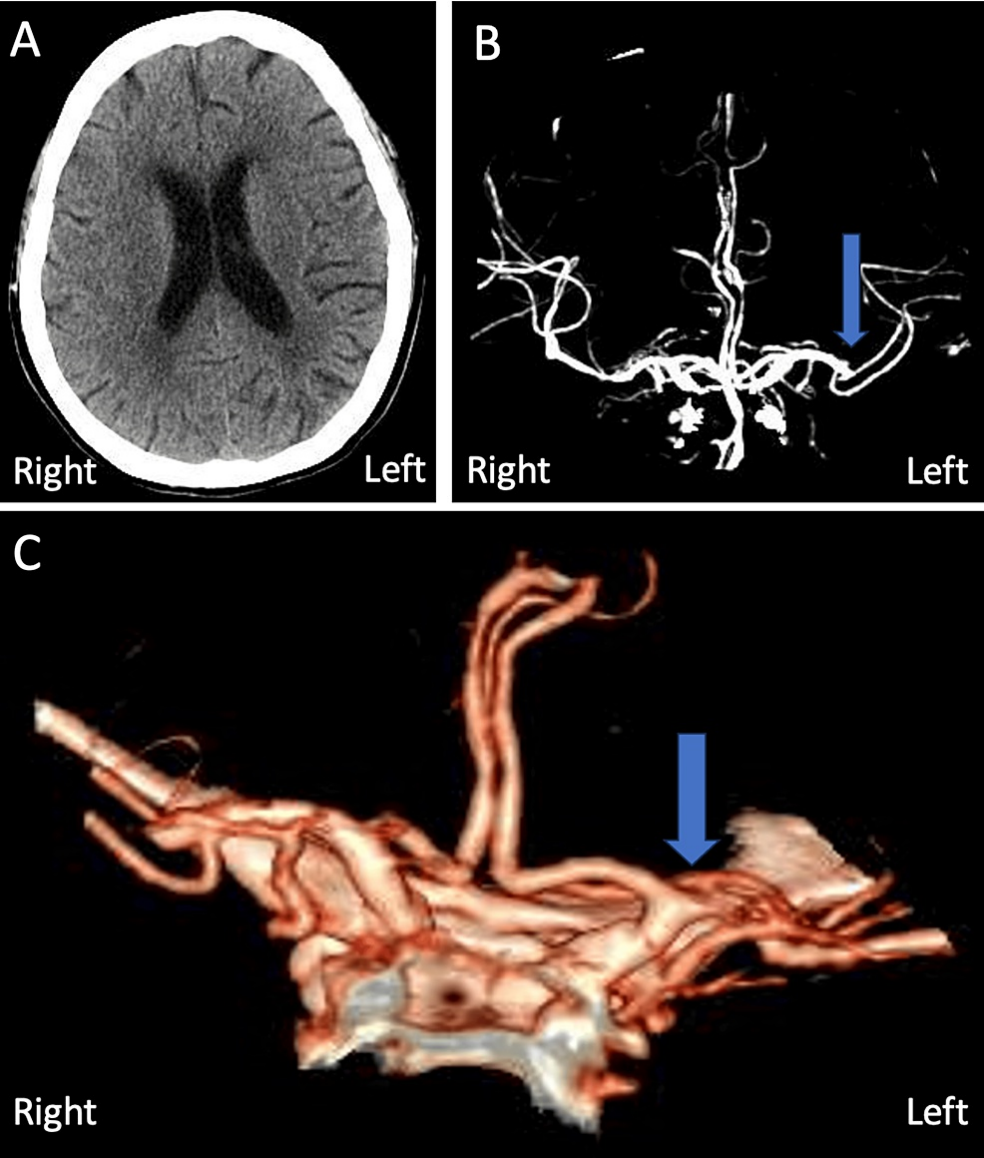
CT images upon initial presentation A) Axial non-contrast image. B) Coronal CT angiography image. C) 3D reconstruction of coronal CT angiography. Blue arrows show the location of the middle cerebral artery occlusion CT: computed tomography

A 68-year-old female with a past medical history of uncontrolled diabetes, hypertension, and hyperlipidemia, presented with a two-week history of multiple 20-30-minute-to-hours-long episodic global aphasia, dysarthria, right facial droop, and right arm weakness. At home, she had been taking clopidogrel 75 mg once daily for an unknown indication. Upon arrival at the emergency room, her NIHSS score was 12, and her blood pressure was 119/73 mmHg. Non-contrast CT showed a chronic-appearing infarct in the left parietal occipital region with mild encephalomalacia and ex-vacuo dilation (Figure 1A). CT angiography (CTA) showed a left-posterior second branch of the middle cerebral artery (M2) occlusion with post-occlusion reconstitution (Figures 1B-1C).

**Figure 2:**
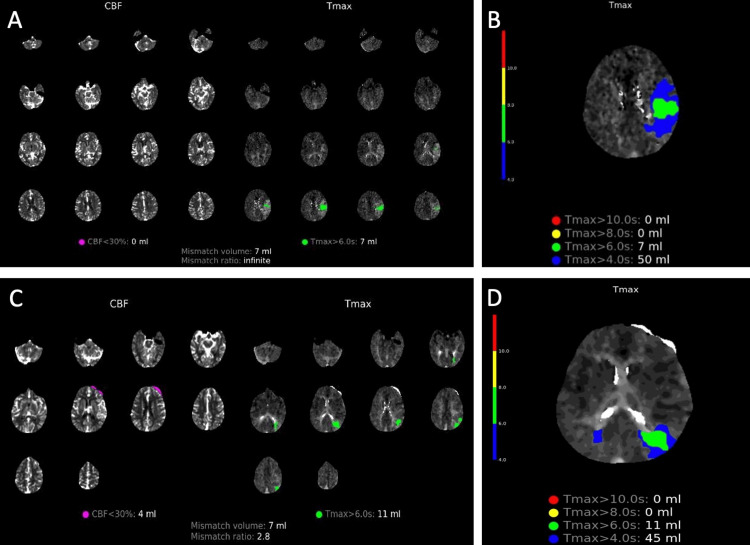
CT perfusion images A) CT perfusion analysis depicting possible core and penumbra at initial presentation. B) Detailed image of CT perfusion at initial presentation with various Tmax values. C) CT perfusion analysis depicting possible core and penumbra on day 11 of admission. D) Detailed image of CT perfusion on day 11 with various Tmax values CT: computed tomography; CBF: cerebral blood flow; Tmax: time-to-maximum

CTP showed no ischemic core but delayed perfusion of 7 ml in Tmax >6 seconds and 50 ml in Tmax >4 seconds (Figures 2A-2B).

**Figure 3:**
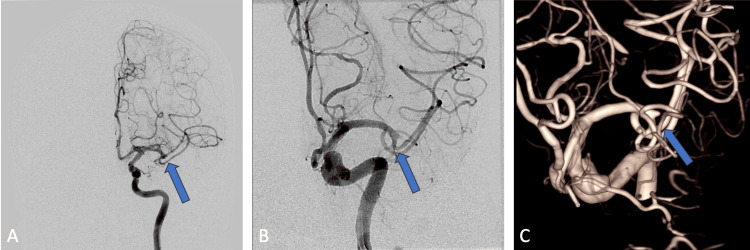
Digital subtraction angiography during attempted thrombectomy A) coronal injection of the left internal carotid artery. B) magnified coronal injection of the left internal carotid artery. C) 3D rendering of the oblique view of the left internal carotid artery injection. Blue arrows show the location of middle cerebral artery occlusion

Due to the M2 occlusion, thrombectomy was attempted by the interventional neurology team by utilizing a RED 43 reperfusion catheter (Penumbra Inc). During the procedure, digital subtraction angiography (DSA) redemonstrated this occlusion with a tapered blunt end in the left middle cerebral artery (Figures 3A-3C).

**Figure 4:**
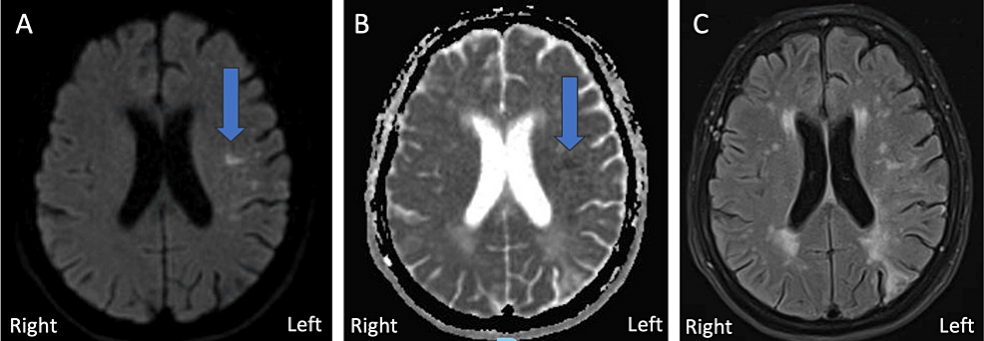
MRI A) Axial diffusion-weighted imaging. B) Axial apparent diffusion coefficient imaging. C) Axial T2 fluid-attenuated inversion recovery imaging. Blue arrows show the location of restriction diffusion with apparent diffusion coefficient correlate MRI: magnetic resonance imaging

Mechanical thrombectomy failed to recanalize the artery and only TICI 0 was achieved. Post-procedure, neurological deficits were observed to be blood pressure-dependent and demonstrated close to complete resolution when the systolic blood pressure (SBP) was maintained above 160 mmHg with vasopressors. Subsequent brain MRI showed acute ischemic infarcts scattered in the left subinsular, frontal, and parietal regions, in the distribution of the left MCA (Figures 4A-4C).

**Figure 5:**
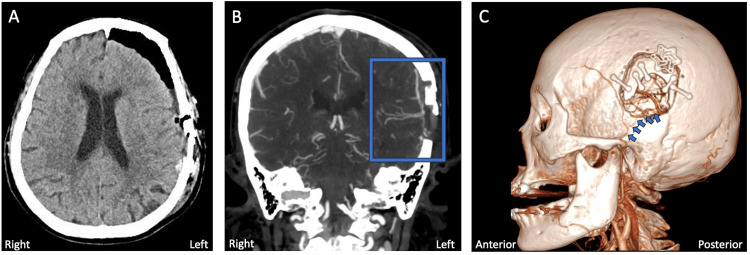
CT head imaging after STA-MCA bypass surgery A) Axial CT non-contrast imaging depicting left hemisphere post-surgical changes. B) Coronal CT angiography depicting anastomosis of the STA with the distal MCA. C) 3D rendering of sagittal CT angiography with patent STA-MCA anastomosis. The blue box focuses on the site of anastomosis; blue arrows track the cranial progression of the superficial temporal artery into the skull CT: computed tomography; STA: superficial temporal artery; MCA: middle cerebral artery

Attempts to wean the patient off from vasopressors were unsuccessful as she became significantly symptomatic with aphasia and right-sided weakness. Considering the area of hypoperfusion on CT perfusion, the decision was made to complete a targeted bypass of that region. On day 10 of admission, under general anesthesia, the patient underwent a posterior frontal craniotomy and STA-MCA bypass to augment the left MCA territory flow (Figures 5A-5B).

**Figure 6:**
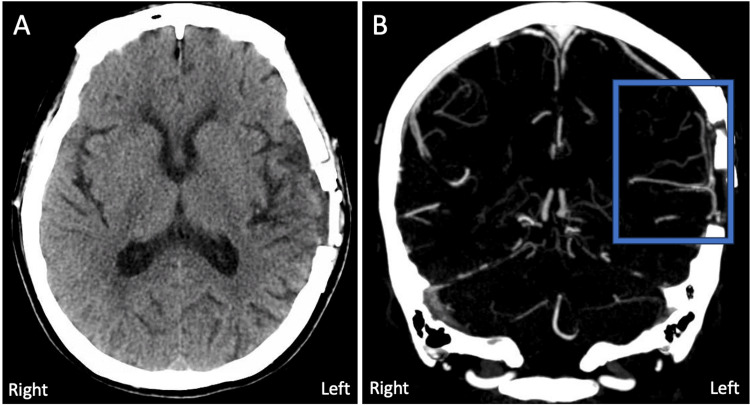
CT head imaging at the 5-month follow-up A) Axial CT non-contrast imaging depicting resolution of postoperative air. B) Coronal CT angiography demonstrating STA-MCA bypass. The blue box focuses on the site of anastomosis CT: computed tomography; STA: superficial temporal artery; MCA: middle cerebral artery

On day 11, the patient had a repeat CTP, showing the evolution of the initial stroke volume (Tmax >6 seconds) but improved total delay perfusion volume from 50 ml to 45 ml when measured with Tmax >4 seconds (Figures 2C-2D). Intravenous vasopressor was switched to oral midodrine, which was later tapered and discontinued. Antiplatelet was converted from clopidogrel to daily aspirin. The patient remained largely asymptomatic and, upon discharge, her NIHSS score was 1 for right arm drift. Her SBP at discharge ranged from 110 to 120 mmHg. At the five-month follow-up, the patient’s family reported mild short-term memory impairment, worsening aphasia, and right-side weakness in the morning when SBP dropped below 100 mmHg. Repeat CT angiography demonstrated the STA-MCA bypass while the non-contrast CT head redemonstrated the site of craniotomy with the resolution of postoperative air (Figure 6). 

Hence, the patient was restarted on 10 mg midodrine to be taken every eight hours. Afterward, the patient and her family reported no additional stroke-like episodes. Her mRs score at 90 days (mRS-90) was 1.

## Discussion

We discuss a case of a patient with blood pressure-dependent stroke symptoms and CTP showing persistent penumbra. After the STA-MCA bypass, the patient tolerated normotension without severe aphasia and arm weakness with an improved NIHSS score from 12 to 1 at discharge with a greatly improved quality of life.

In recent years, patients selected for EC-IC bypass have often presented with small ischemic stroke symptoms secondary to a large vessel occlusion that failed endovascular recanalization with a high ratio of delay perfusion mismatch on CTP [12-14]. Although the reduced ischemic penumbra, in this case, was only 5 milliliters in volume before discharge (Tmax >4 sec from 50 ml to 45 ml), the extent of improvement was comparable to the previously reported volume of 2.63 ± 0.93 milliliters, which was measured by the reduced mean transient time >145% compared to the contralateral side [15]. Interestingly, a long-term follow-up study of EC-IC bypass showed a continuously reduced volume of perfusion mix-matching and total delayed CTP at discharge and, later, at six-month follow-ups with a reported recurrent rate of ischemic stroke of 4.7% [16]. This finding is lower than the historically reported average of 10% at the six-month follow-up after strokes due to large-artery atherosclerosis [17]. Given our findings, we hope to revive interest in designing a future prospective randomized EC-IC bypass trial in patients with pressure-dependent stroke symptoms.

## Conclusions

We presented a case of a patient with blood pressure-dependent stroke symptoms who failed endovascular thrombectomy but whose condition subsequently improved with STA-MCA bypass. The patient was selected for the procedure based on detailed interpretations of CTP software, rather than conventional SPECT and TCD modalities. Our study demonstrates how candidates for STA-MCA bypass can be selected in a cost-effective manner. Overall, we propose that CTP and blood pressure-dependent symptoms are pragmatic and feasible markers to help screen potential beneficiaries of the EC-IC bypass procedure.
